# Analysis and Prediction of Corrosion of Refractory Materials by Potassium during Biomass Combustion-Thermodynamic Study

**DOI:** 10.3390/ma11122584

**Published:** 2018-12-18

**Authors:** Ying Zhao, Guishi Cheng, Fei Long, Lu Liu, Changqing Dong, Xiaoqiang Wang, Jin Zhao

**Affiliations:** 1 National Engineering Laboratory for Biomass Power Generation Equipment, School of the Renewable Energy, North China Electric Power University, Beijing 102206, China; 51101890@ncepu.edu.cn (G.C.); 51102247@ncepu.edu.cn (L.L.); cqdong@ncepu.edu.cn (C.D.); energy@ncepu.edu.cn (X.W.); 2 Department of Mechanical and Material Engineering, Queen’s University, Kingston, ON K7L 3N6, Canada; long.fei@queensu.ca; 3 State Grid Energy Conservation Services CO., Ltd., Beijing 100056, China; zhaojin@sgecs.sgcc.com.cn

**Keywords:** corrosion, refractory material, thermodynamic, potassium salt

## Abstract

As a kind of renewable resource, biomass has been used more and more widely, but the potassium contained in biomass can cause corrosion of the refractory. For a better understanding of corrosion thermodynamic mechanisms, the five components of common refractory materials (magnesium chrome spinel MgO·Cr_2_O_3_, magnesium aluminum spinel MgO·Al_2_O_3_, Al_2_O_3_, MgO, and Cr_2_O_3_) with potassium salts (K_2_CO_3_, K_2_SO_4_, and KCl) under high-temperature were studied by using the FactSage^TM^ 7.0 software. Thermodynamic calculation results indicate that MgO is the best corrosion resistance of the five components of refractory materials. Based on the obtained results, the corrosion experiments in the laboratory were carried out (muffle furnace or high-temperature tube furnace) for corrosion reaction of KCl and MgO. The chemical compositions of the corroded samples were analyzed by X-ray diffraction (XRD). Under laboratory conditions (600–1200 °C), no corrosion products have been observed in the high-temperature corrosion experiments. The result indicates that to prevent the corrosion processes, refractories should contain as much MgO as possible.

## 1. Introduction

The total amount of biomass accumulated in the world every year is equivalent to 10 times of the total energy consumption of the year. Biomass is the fourth energy after coal, oil, and natural gas, and contributes 14% to the world’s primary energy consumption [1,2]. Biomass has the characteristic of near-zero carbon dioxide emissions. Therefore, the development and utilization of biomass energy is an important way to optimize energy structure, alleviate the contradiction between energy supply and demand, protect the environment and reduce greenhouse gas emissions. As one of the important renewable energy sources, biomass energy occupies an important position in energy system [3]. In recent years, China has accelerated the adjustment of its energy structure, actively promoted the development and utilization of biomass energy, and biomass power generation has been flourishing. In coal K primarily exists in the format of feldspar (K_2_O·Al_2_O_3_·6SiO_2_) while the content of activated K species (e.g., K_2_SO_4_, KCl, K_2_CO_3_) is negligible [4,5]. However, it is very different in biomass. As it has a very high ratio of oxygen, a part of potassium is combined with a strong oxygen-containing functional group, and some other parts are present in the inner mineral or combined with the organic functional group. Since the potassium content of biomass is much higher than that of coal, high levels of potassium compounds, especially potassium chloride, can cause severe corrosion of boilers during combustion.

Many studies have focused on the mechanism of potassium release during biomass combustion/gasification/pyrolysis [6,7,8,9]. J. M. Johansen et al. [10] reported that Cl was the main facilitator for K release through sublimation of KCl at the reaction temperature above 700–800 °C. H. Kassman et al. [11] also reported that Cl could facilitate the release of K by the formation of KCl (g) in the biomass combustion at high temperatures. In addition, the ash agglomeration behavior caused by potassium during biomass combustion/pyrolysis/gasification is also the focus of researchers [12,13,14,15]. H. Zhan et al. [16] suggested that coal ash is a potential additive for not only fixing potassium, but also increasing the temperatures of biomass ash fusion. The effect of potassium compounds on the corrosion of equipment in biomass is mainly focused on the superheater metal materials [17,18,19,20]. Nevertheless, up to now, only a few researches have focused on the mechanism of corrosion of refractory by biomass ash [21]. It is very limited that there are thermodynamic studies on the corrosion of refractory by active potassium in biomass combustion are limited.

Since the corrosion behavior of potassium on refractories during biomass combustion is greatly affected by temperature and potassium salt type, this study mainly investigates different combustion temperatures (600, 700, 800, 900, 1000, 1100, and 1200 °C). Different kinds of active potassium (K_2_CO_3_, K_2_SO_4_, and KCl) corrode five components of common refractory materials (magnesium chrome spinel MgO·Cr_2_O_3_, magnesium aluminum spinel MgO·Al_2_O_3_, Al_2_O_3_, MgO, and Cr_2_O_3_). The FactSage^TM^ 7.0 software was used to simulate the interaction of the five components with three kinds of potassium salts based on the established calculation model and predict the phase formation when thermodynamic equilibrium was reached. The Gibbs free energy of each reaction under different conditions are calculated by using the FactSage^TM^ 7.0 software. We systematically analyzed the possibility of reacting five components of common refractory materials with three kinds of potassium salts and the mineral phase of corrosion products. We obtained the direction and limit of reaction in the multi-reaction system of refractory by potassium corrosion process and the affection of corrosion by temperature and potassium salt type. The goal of this paper is to provide guidance for the selection and optimization of biomass boiler refractories.

## 2. Thermodynamic Calculations

Thermodynamic calculations were carried out using the version 7.0 of the FactSage™ software package (GTT Technologies, Aachen, Germany and Thermfact/CRCT, Montreal, QC, Canada), because it is one of the largest fully integrated databases in the field of chemical thermodynamics. FactSage™ 7.0 software contains several thermodynamic databases, including a database of FactPS (the FactSage™ 7.0 contains 6872 kinds of compounds) evaluation and optimization of hundreds of metal solutions, oxide liquid phase and solid phase solutions, hydrazine, molten salt, aqueous solutions and other solution databases. The FactSage™ software is based on the system’s minimum Gibbs free energy and provides information on the phases formed, their proportions and composition, the individual activities of each chemical component, and the thermodynamic properties of various compositions, pressures, and temperatures [22]. Therefore, FactSage™ software can conduct the thermodynamic calculation of the corrosion of refractory by potassium salt. In this study, the Equilib module was used which is adapted to multi-component systems like potassium compounds and refractory materials.

In this paper, three kinds of oxide components commonly used in refractories and two kinds of spinels synthesized by them are selected as research objects. The three oxides are Al_2_O_3_, MgO, and Cr_2_O_3_, and the two spinels are MgO·Cr_2_O_3_ and MgO·Al_2_O_3_. In order to investigate the corrosion mechanism of potassium compounds on the five components of refractory materials, three potassium salts K_2_CO_3_, K_2_SO_4_, and KCl were selected. This is because active potassium is present in the solid and gas phases in the form of these three potassium salts during biomass combustion.

1 mol of the five components of refractory materials and three potassium salts were used as input data for the calculation. The Equilib module was used to calculate the change of Gibbs free energy in the corrosion of five components of refractory materials by three potassium salts. The trend of reaction was analyzed. The larger the negative value of Gibbs free energy, the greater the reaction trend. Moreover, corrosion products at different temperatures were obtained, and the chemical reaction equations that may occur during the corrosion process were inferred. All simulations were performed with temperatures varying from 600 to 1200 °C in steps of 100 °C at 1 atm pressure.

## 3. Thermodynamic Experimental Procedure

A total of three sets of experiments were performed at different temperatures, 600, 1000, and 1200 °C. The corrosion test at 600 °C is controlled by a control box type muffle furnace (GW-300C China Coal Research Institute, Beijing, China). Since the maximum temperature of the muffle furnace is less than 1000 °C, the corrosion experiments at 1000 °C and 1200 °C were performed by high-temperature tube furnace (TF-1700C Beijing MeiCheng Scientific Instrument Co., Ltd., Beijing, China). Control box type resistance muffle furnace, with a double furnace ceramic fiber structure and a modular control system, can automatically raise temperature and maintain the constant temperature, and has accurate stable and reliable temperature control. The high-temperature tube furnace adopts a high-temperature corundum tube, and the temperature can be controlled by the temperature controller, which allows the temperature to vary between room temperature and 1700 °C.

KCl and MgO samples (molar ratio of 1) were placed in muffle furnace or high-temperature tube furnace. After warming to a specified temperature, the furnace stayed in standby mode for 4 h. When the experiment was completed and the tube furnace was cooled to room temperature, experiment reaction products were analyzed by XRD (Shimadzu, Kyoto, Japan) and Jade 5.0 software (Materials Data Ltd., Livermore, CA, USA).

## 4. Results and Discussion

### 4.1. Corrosion Reaction of K_2_CO_3_ and the Five Components of Refractory Materials

**Figure 1:**
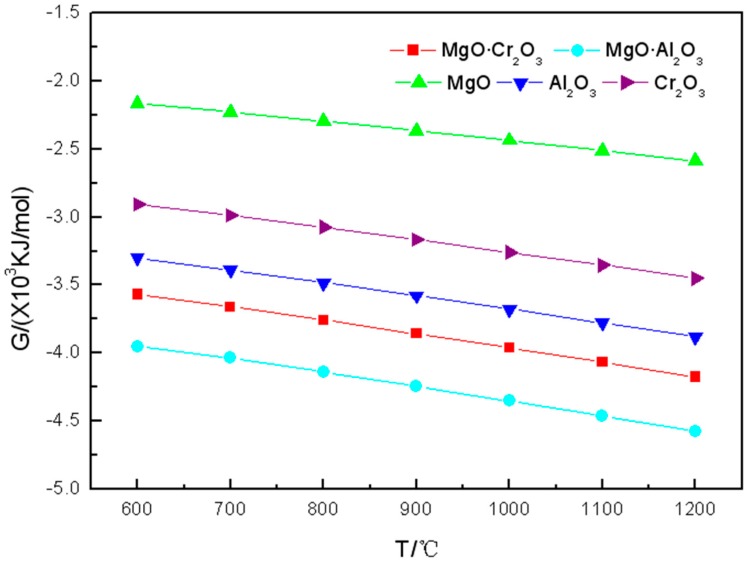
Gibbs free energy of the reaction between K_2_CO_3_ and the five components of refractory materials in the 600–1200 °C temperature range.

As can be seen from Figure 1, in the range of 600 to 1200 °C, the Gibbs free energy of the reactions between each of the five components of refractory materials and K_2_CO_3_ are all negative. The Gibbs free energy becomes more negative gradually with the increasing of the temperature which indicates that the higher the temperature, the greater the tendency of the refractory to be corroded by K_2_CO_3_.

Corrosion products and reaction equations of K_2_CO_3_ reacting with five components of refractory materials are shown in Table 1. It can be seen from Table 1 that the two oxides (Al_2_O_3_ and Cr_2_O_3_) and the two spinels are corroded by potassium carbonate at 600 °C. The reactions in which Cr_2_O_3_ and MgO·Cr_2_O_3_ are corroded by K_2_CO_3_ at 600–1200 °C are

K_2_CO_3_ + 1/2 Cr_2_O_3_ + 3/4O_2_ = K_2_CrO_4_ + CO_2_ (1)

K_2_CO_3_ + 1/2 MgO·Cr_2_O_3_ + 3/4O_2_ = 1/2MgO + K_2_CrO_4_ + CO_2_ (2)

The reaction product of Al_2_O_3_ and MgO·Al_2_O_3_ corroded by K_2_CO_3_ and the degree of reaction are related to temperature. At 600 °C, 0.3416 mol of Al_2_O_3_ is corroded by K_2_CO_3_ to form K_2_ Al_12_O_19_ through reaction (3), and 0.6584 mol of Al_2_O_3_ is corroded by K_2_CO_3_ to form KAlO_2_ through reaction (4). At 700–1200 °C, Al_2_O_3_ is corroded by K_2_CO_3_ through reaction (4), and 1 mol of Al_2_O_3_ is completely corroded to form 2 mol of KAlO_2_.

The two reactions in which Al_2_O_3_ is corroded by K_2_CO_3_ are

K_2_CO_3_ + 6Al_2_O_3_ = K_2_Al_12_O_19_ + CO_2_ (3)

K_2_CO_3_ + Al_2_O_3_ = 2KAlO_2_ + CO_2_ (4)

At 600 °C, only 0.0608 mol of MgO·Al_2_O_3_ is corroded to form KAlO_2_ and MgO, while at 700–1200 °C, 1 mol of MgO·Al_2_O_3_ is completely corroded, as described by reactions (5).

K_2_CO_3_ + MgO·Al_2_O_3_ = 2KAlO_2_ + MgO + CO_2_ (5)

Furthermore, it can be seen from the table that the amount of corrosion products in which MgO reacted with K_2_CO_3_ is less than 10^−4^ mol. However, it can be seen from Figure 1 that the Gibbs free energy of the reaction between K_2_CO_3_ and MgO is negative. This is because the calculation results of FactSage^TM^ 7.0 showed that K_2_CO_3_ will decompose at 600–1200 °C to produce KO_2_, K_2_O_2_, and K_2_O. This explains why the Gibbs free energy of MgO reacts with K_2_CO_3_ is negative. Therefore, MgO is the best corrosion resistance of the five components of refractory materials.

### 4.2. Corrosion Reaction of K_2_SO_4_ and the Five Components of Refractory Materials

**Figure 2:**
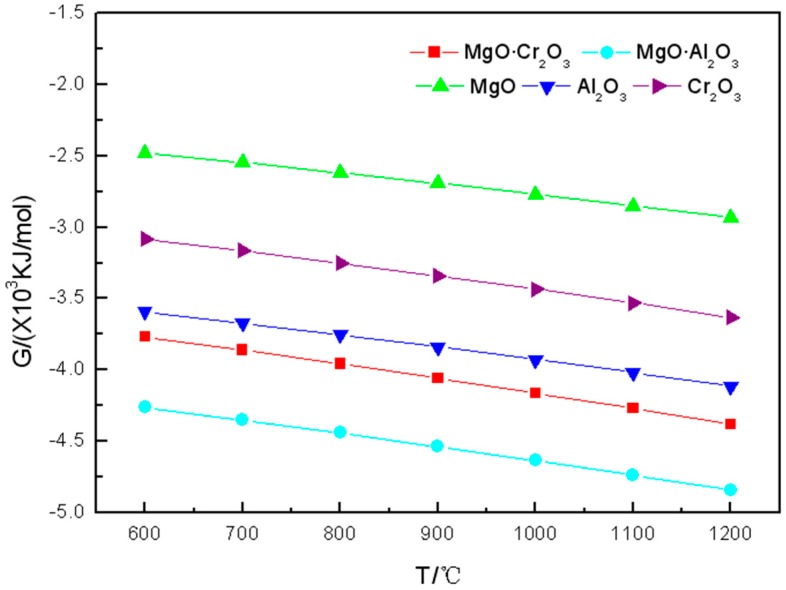
Gibbs free energy of the reaction between K_2_SO_4_ and the five components of refractory materials in the 600–1200 °C temperature range.

It can be seen from Figure 2 that the tendency of Gibbs free energy change of the five components of refractory materials reacted with K_2_SO_4_ in the range of 600 to 1200 °C is consistent with the one of reactions between the five components of refractory materials and K_2_CO_3_. Two spinels (MgO·Cr_2_O_3_ and MgO·Al_2_O_3_) are more strongly corroded by K_2_SO_4_, while MgO has the least tendency to be corroded by K_2_SO_4_.

From Table 2, we can find that at 600–900 °C, the five components of refractory materials are not corroded by K_2_SO_4_. However, when the temperature further increased to 1000 °C, a small amount of Cr_2_O_3_ (0.0001 mol) is corroded by K_2_SO_4_ to form K_2_CrO_4_. As the temperature increases, more Cr_2_O_3_ is corroded by K_2_SO_4_ through reactions (6)

K_2_SO_4_ + 1/2 Cr_2_O_3_ + 3/4O_2_ = K_2_CrO_4_ + SO_3_ (6)

Similarly, when the temperature rises to 1100 °C, 0.004 mol of Al_2_O_3_ and 0.00005 mol of MgO·Cr_2_O_3_ are corroded by K_2_SO_4_ to form KAl_9_O_14_ and K_2_CrO_4_, respectively. As the temperature increases, more Al_2_O_3_ and MgO·Cr_2_O_3_ are corroded by K_2_SO_4_, and the reactions of Al_2_O_3_ and MgO·Cr_2_O_3_ by K_2_SO_4_ are

K_2_SO_4_ + 9Al_2_O_3_ = 2KAl_9_O_14_ + SO_3_ (7)

K_2_SO_4_ + 1/2 MgO·Cr_2_O_3_ + 3/4O_2_ = K_2_CrO_4_ + 1/2MgO + SO_3_ (8)

It can also be seen from the table that the amount of corrosion products in which MgO or MgO·Al_2_O_3_ reacted with K_2_SO_4_ is less than 10^−4^ mol.

In summary, Cr_2_O_3_ is most susceptible to be corroded by K_2_SO_4_, followed by Al_2_O_3_ and MgO·Cr_2_O_3_, while MgO and MgO·Al_2_O_3_ are the most difficult ones to be corroded by K_2_SO_4_.

### 4.3. Corrosion Reaction of KCl and the Five Components of Refractory Materials

**Figure 3:**
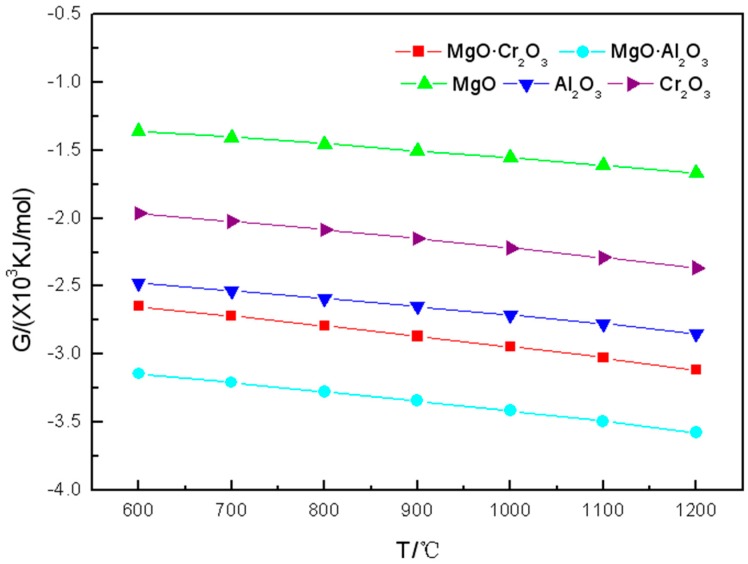
Gibbs free energy of the reaction between KCl and the five components of refractory materials in the 600–1200 °C temperature range.

As can be seen from Figure 3, in the range of 600–1200 °C, the tendency of Gibbs free energy change of the five components of refractory materials reacted with KCl is consistent with the one of the reactions between the five components of refractory material and K_2_CO_3_ or K_2_SO_4_.

It can be seen from Table 3 that at 600–900 °C, the five components of refractory materials are not corroded by KCl. However, when the temperature further increased to 1000 °C, a small amount of Al_2_O_3_ (0.0009 mol) and Cr_2_O_3_ (0.00015 mol) are corroded by KCl to form KAl_9_O_14_ and K_2_CrO_4_, respectively. The increase in temperature causes more Al_2_O_3_ and Cr_2_O_3_ to be corroded by KCl. The reaction equations are

2KCl + 9Al_2_O_3_ + 1/2O_2_ = 2KAl_9_O_14_ + Cl_2_ (9)

4KCl + Cr_2_O_3_ + 5/2O_2_ = 2K_2_CrO_4_ + 2Cl_2_ (10)

When the temperature rises to 1100 °C, 0.00005 mol of MgO·Cr_2_O_3_ is corroded by KCl to form K_2_CrO_4_, and the reaction of MgO·Cr_2_O_3_ by KCl is

4KCl + MgO·Cr_2_O_3_ + 5/2O_2_ = 2K_2_CrO_4_ + MgO + 2Cl_2_ (11)

It can also be seen from the table that the amount of corrosion products in which MgO or MgO·Al_2_O_3_ reacted with KCl is less than 10^−4^ mol.

In summary, Al_2_O_3_ and Cr_2_O_3_ are most susceptible to corrosion by KCl, followed by MgO·Cr_2_O_3_, and MgO and MgO·Al_2_O_3_ are the most difficult to be corroded by KCl.

It is worth noting that, as can be seen from Figure 1, Figure 2 and Figure 3, the Gibbs free energy of MgO·Al_2_O_3_ reacting with the three potassium salts is the smallest. However, it can be seen from the corrosion products calculated from FactSage^TM^ 7.0 that MgO·Al_2_O_3_ is the least susceptible to potassium salt in the five refractory components except MgO. This also shows that the reaction tendency of the corrosion reaction is the only one that can be obtained by calculating the Gibbs free energy. The estimation of difficulty of the corrosion reaction needs to be obtained by considering the products of Table 1, Table 2 and Table 3 regarding the corrosion of the refractory component by the potassium salts.

### 4.4. Thermodynamic Experiment

As can be seen from the results calculated by the FactSage^TM^ 7.0 software, MgO has the best corrosion resistance and is most suitable for refractory materials resistant to potassium salt corrosion. KCl is the most abundant potassium salt in biomass combustion process, so it is necessary to investigate the thermodynamic experiment of corrosion of MgO by KCl.

**Figure 4:**
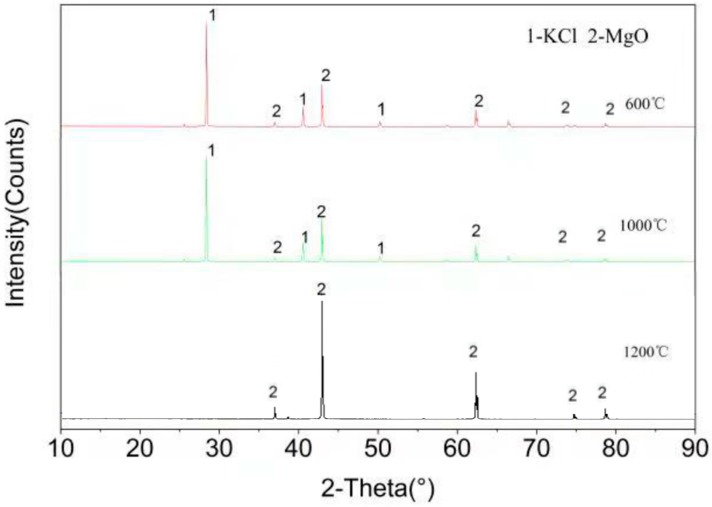
XRD patterns of the products of the reaction between MgO and KCl.

Figure 4 shows the XRD spectra of samples after the test of high temperature corrosion of KCl and MgO at three temperatures of 600, 1000, and 1200 °C. As can be seen from the figure, only MgO and KCl are detected in the sample at the temperatures of 600 °C and 1000 °C, and no new substance is formed. Only MgO is detected in the sample at the temperature of 1200 °C, which is consistent with the results from the modulation by Factsage software. Under the condition of high temperature 1200 °C, KCl is in the form of gas. It shows that MgO has good resistance to KCl corrosion at 1200 °C, which is consistent with the results calculated by FactSage^TM^ 7.0 software (see Table 3).

## 5. Conclusions

From the viewpoint of thermodynamics, this study focused on the Gibbs free energy and the mineral phase of corrosion products of potassium salts corrosion to the five components of refractory materials using thermodynamic calculation method. According to the calculation results, corrosion experiments in the 600–1200 °C temperature range were carried out.

From the calculation results using FactSage^TM^ 7.0 software, it can be found that the MgO has the best resistance to the potassium salts corrosion, followed by MgO·Al_2_O_3_ and MgO·Cr_2_O_3_, and Al_2_O_3_ and Cr_2_O_3_ have the worst resistance to the potassium salts corrosion. The new phases were KAlO_2_ and K_2_Al_12_O_19_ which appeared as the result of chemical reaction between Al_2_O_3_ and K_2_CO_3_, while Al_2_O_3_ reacts with K_2_CO_3_ or KCl to form KAl_9_O_14_. K_2_CrO_4_ was also a new phase in Cr_2_O_3_ or MgO·Cr_2_O_3_ as the product of the reaction with the three potassium salts. No corrosion products were found in the reaction of MgO with the three potassium salts. Finally, the results obtained from the FactSage^TM^ 7.0 calculations agrees with XRD analysis. The negative effects of corrosion problems caused by potassium salt can be limited by using refractories contained as much MgO as possible.

## Figures and Tables

**Table 1:** Corrosion products and reaction equations of K_2_CO_3_ reacting with the five components of refractory materials.

Species	Temperature (°C)	
	600	700–1200
Al_2_O_3_	1.3168 mol KAlO_2_, 0.0569 mol K_2_Al_12_O_19_	2 mol KAlO_2_
	Equations (3) and (4)	Equation (4)
MgO	-	
Cr_2_O_3_	1 mol K_2_CrO_4_	
	Equation (1)	
MgO·Cr_2_O_3_	1 mol K_2_CrO_4_, 0.5mol MgO	
	Equation (2)	
MgO·Al_2_O_3_	0.1215 mol KAlO_2_, 0.0608 mol MgO	2 mol KAlO_2_, 1 mol MgO
	Equation (5)	Equation (5)

“-”: The amount of corrosion products is less than 10^−4^ mol.

**Table 2:** Corrosion products and reaction equations of K_2_SO_4_ reacting with the five components of refractory materials.

Species	Temperature (°C)			
	600–900	1000	1100	1200
Al_2_O_3_	-		0.0009 mol KAl_9_O_14_	0.0062 mol KAl_9_O_14_
			Equation (7)	Equation (7)
MgO	-			
Cr_2_O_3_	-	0.0002 mol K_2_CrO_4_	0.0015 mol K_2_CrO_4_	0.0076 mol K_2_CrO_4_
		Equation (6)	Equation (6)	Equation (6)
MgO·Cr_2_O_3_	-		0.0001 mol K_2_CrO_4_	0.0008 mol K_2_CrO_4_
			Equation (8)	Equation (8)
MgO·Al_2_O_3_	-			

“-”: The amount of corrosion products is less than 10^−4^ mol.

**Table 3:** Corrosion products and reaction equations of KCl reacting with the five components of refractory materials.

Species	Temperature (°C)			
	600–900	1000	1100	1200
Al_2_O_3_	-	0.0002 mol KAl_9_O_14_	0.0004 mol KAl_9_O_14_	0.0009 mol KAl_9_O_14_
		Equation (8)	Equation (8)	Equation (8)
MgO	-			
Cr_2_O_3_	-	0.0003 mol K_2_CrO_4_	0.0005 mol K_2_CrO_4_	0.0006 mol K_2_CrO_4_
		Equation (10)	Equation (10)	Equation (10)
MgO·Cr_2_O_3_	-		0.0001 mol K_2_CrO_4_	0.0002 mol K_2_CrO_4_
			Equation (11)	Equation (11)
MgO·Al_2_O_3_	-			

“-”: The amount of corrosion products is less than 10^−4^ mol.
